# Cancers not detected in one-view breast tomosynthesis screening—characteristics and reasons for non-detection

**DOI:** 10.1007/s00330-024-11278-2

**Published:** 2024-12-20

**Authors:** Kristin Johnson, Debra M. Ikeda, Ingvar Andersson, Sophia Zackrisson

**Affiliations:** 1 Radiology Diagnostics, Department of Translational Medicine, Lund University, Skåne University Hospital, Malmö, Sweden; 2 Department of Imaging and Physiology, Skåne University Hospital, Malmö, Sweden; 3 Department of Radiology, Stanford University School of Medicine, Stanford, CA USA; 4 Unilabs Mammography Unit, Skåne University Hospital, Malmö, Sweden

**Keywords:** Breast, Screening, Tomosynthesis, Adults, Mammography

## Abstract

**Objectives:**

Limited understanding exists regarding non-detected cancers in digital breast tomosynthesis (DBT) screening. This study aims to classify non-detected cancers into true or false negatives, compare them with true positives, and analyze reasons for non-detection.

**Materials and methods:**

Conducted between 2010 and 2015, the prospective single-center Malmö Breast Tomosynthesis Screening Trial (MBTST) compared one-view DBT and two-view digital mammography (DM). Cancers not detected by DBT, i.e., interval cancers, those detected in the next screening round, and those only identified by DM, underwent a retrospective informed review by in total four breast radiologists. Reviewers classified cancers into true negative, false negative, or non-visible based on both DBT and DM findings and assessed radiographic appearances at screening and diagnosis, breast density, and reasons for non-detection. Statistics included the Pearson *X*^2^ test.

**Results:**

In total, 89 cancers were not detected with DBT in the MBTST; eight cancers were solely in the DM reading mode, 59 during subsequent DM screening rounds, and 22 interval cancers. The proportion of cancers classified as false negative was 25% (22/89) based on DBT, compared with 18% (14/81) based on DM screening. The primary reason for false negatives was normal-appearing density, 50% (11/22). False negatives exhibited lower rates of high breast density, 36% (8/22), compared with true positives, 61% (78/129), *p* = 0.04, and spiculated densities were less frequent in false negatives, 41% (9/22) compared with true positives, 68% (88/129), *p* = 0.01.

**Conclusion:**

False negatives in one-view DBT screening commonly presented with spiculated features, but less frequently than true positives, and were missed or misinterpreted due to benign appearances.

**Key Points:**

***Question*** *Cancers not detected in digital breast tomosynthesis screening, including false negatives, remain partly unexplored.*

***Findings*** *The most common reason behind false-negative cancers in a large screening trial was a normal-appearing density.*

***Clinical relevance*** *Recognizing the factors contributing to false negative findings in digital breast tomosynthesis screening is essential to further improve cancer detection.*

**Graphical Abstract:**

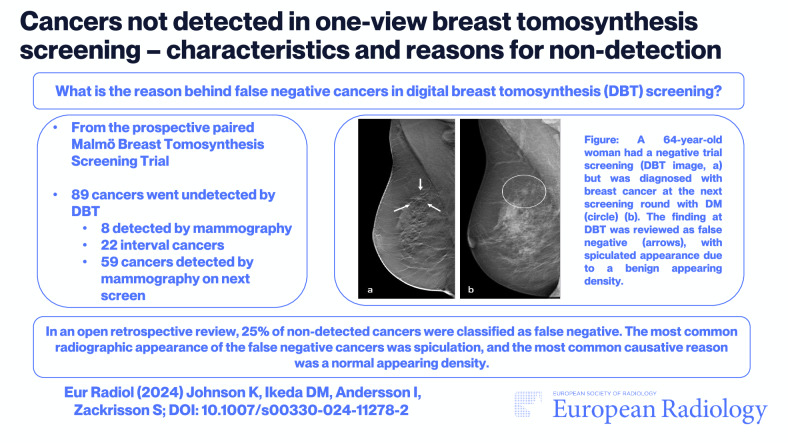

## Introduction

Screening utilizing digital breast tomosynthesis (DBT) has demonstrated enhanced breast cancer detection compared with digital mammography (DM), as evidenced by both prospective trials and retrospective studies [1]. Cancers detected through DBT exhibit similar or even more favorable characteristics than those identified through DM screening [2–4]. Additionally, research indicates that cancers detected in DBT screening display a higher proportion of spiculated radiographic features compared with DM screening [2, 5].

False negative cancers, i.e., cancers not detected at the screening examination but visible retrospectively, are an inevitable consequence of screening. Not much is known about false negative cancers in DBT screening, but results so far indicate similar proportions of true and false negative cancers between DBT and DM [6]. Insights into false negative cancers and other cancers not detected in DBT screening can help improve image interpretation and further increase cancer detection in the future.

The paired Malmö Breast Tomosynthesis Screening Trial (MBTST) compared one-view DBT with standard two-view DM. The trial showed an increased cancer detection [7], lower odds of interval cancer [8] albeit with a slightly higher false positive recall rate compared with DM [9]. However, cancers not detected in the trial despite the more sensitive method DBT, and specifically false negative cancers, remain partly unexplored. Thus, the aim of this study was to classify cancers not detected with DBT in the MBTST into true or false negatives in an informed consensus review and to assess radiographic appearances, breast density, reasons for non-detection, and tumor characteristics of these cancers. Furthermore, the aim was also to compare radiographic appearances and tumor characteristics in non-detected cancers with cancers detected with DBT in the MBTST.

## Materials and methods

This study constitutes a retrospective review of cancers in the MBTST, a population-based paired trial conducted in the city of Malmö, Sweden. The trial targeted women eligible for screening according to the Swedish screening program, age ranges of 40 to 74 years and invited them randomly between January 2010 and February 2015. Participating women gave their written informed consent and the study protocol received approval from the Ethics committee at Lund University (2009/770). All women underwent one-view DBT in the mediolateral oblique view and two-view DM encompassing mediolateral oblique and craniocaudal views, all performed at a single screening occasion. The imaging equipment used was a wide-angle (50°) Mammomat Inspiration, manufactured by Siemens Healthineers. Image interpretation was conducted via two separate reading modes; the DBT reading mode and the DM reading mode, each comprising three sequential reading steps. In the DBT reading mode, step 1 is one-view DBT, step 2 is the addition of current craniocaudal DM images, and step 3 is the addition of previous screening DM images (if any). In the DM reading mode, step 1 is current images, step 2 is the addition of previous screening DM images (if any), and step 3 is density assessment according to Breast Imaging and Reporting Data System (BI-RADS) 4th edition. Reading procedures have been described in more detail previously [7]. The trial was registered at ClinicalTrials.gov, NCT01091545.

### Cancers not detected by DBT

Cancers that were detected only in the DM reading mode, along with interval cancers and those detected during the first subsequent DM screening round after the MBTST, were defined as non-detected cancers in this study, representing potential false negative cancers. Breast cancer cases were ascertained through cross-link with the Swedish cancer registry South. Interval cancers were defined as cancers diagnosed after a negative screening in the MBTST but before the next scheduled screening round. In cases of multifocal or bilateral cancers at diagnosis, the largest invasive cancer was included in this study.

### True-positive cancers

Cancers detected in the DBT reading mode during the MBTST, representing true positives, were used for comparison. The radiographic appearances of those cancers have been evaluated previously, based on the dominant appearance described in the radiology reports, and in unclear cases by review (among them I.A. and S.Z.) [7].

### Retrospective review of images

Images of cancers undetected by DBT underwent retrospective review by two to three members out of a group of four breast radiologists (among them I.A., D.I., and S.Z.) whose experience ranges from 8 to 50 years, with a median of 21 years. The review process was open, granting access to all previous images and reports, and all clinical information was available upon request from the reviewers. The reviewers classified the cancers as true negative not visible (not visible or very discrete findings at previous screening or mammographically occult, i.e., not visible at screening nor diagnostic DM or DBT examination), true negative minimal sign (non-specific or not sufficient to recall for assessment), or false negative missed/misinterpreted (cancer visible at previous screening, suggestive of cancer or sufficient to recall for assessment, but missed/misinterpreted by radiologists), modified according to Houssami et al [10]. The classification was separately assessed both in relation to DBT and DM at screening in the MBTST. Furthermore, the reviewers evaluated the dominant radiographic appearance of the cancers at the time of DM at diagnosis and DBT at screening across seven categories; spiculated density, circumscribed/indistinct density, calcifications, architectural distortion, asymmetry, other or non-visible. Additionally, the reviewers assessed the reasons for non-detection: too small finding, dense tissue, busy breast, normal-appearing density, normal-appearing calcifications, or post-surgical changes. The retrospective review did not include cancers detected with DBT and not DM, but the radiographic appearances of those cancers have been previously evaluated and were used for comparison [2, 9].

### Tumor characteristics and breast density

Breast cancer size (mm) and histological type (invasive carcinoma of no special type, invasive lobular carcinoma, tubular carcinoma, or ductal carcinoma in situ) were assessed through pathology reports. St Gallen molecular subtypes were determined based on estrogen and progesterone receptor, HER2, and Ki67 statuses from pathology reports. Breast density was categorized into two groups: low density (BI-RADS 4th edition 1-2) and high (3–4) density.

### Statistics

Descriptive methods (numbers and percentages) were utilized to analyze and present data concerning non-detected and true-positive cancers. The Pearson *X*^2^ test was employed for descriptive comparisons between high and low breast density, spiculated and not spiculated radiographic appearance, invasive and ductal carcinoma in situ cancers, and luminal A-like and non-luminal A-like subtypes in false negative compared with true-positive cancers. The false-negative rate in DBT screening was defined as the number of false-negative cancers per 1000 screened women, with a corresponding 95% confidence interval calculated using Sample Size Calculators [11]. Other statistical analyses were performed using IBM SPSS Statistics version 29.0.0.0 software.

## Results

**Fig. 1:**
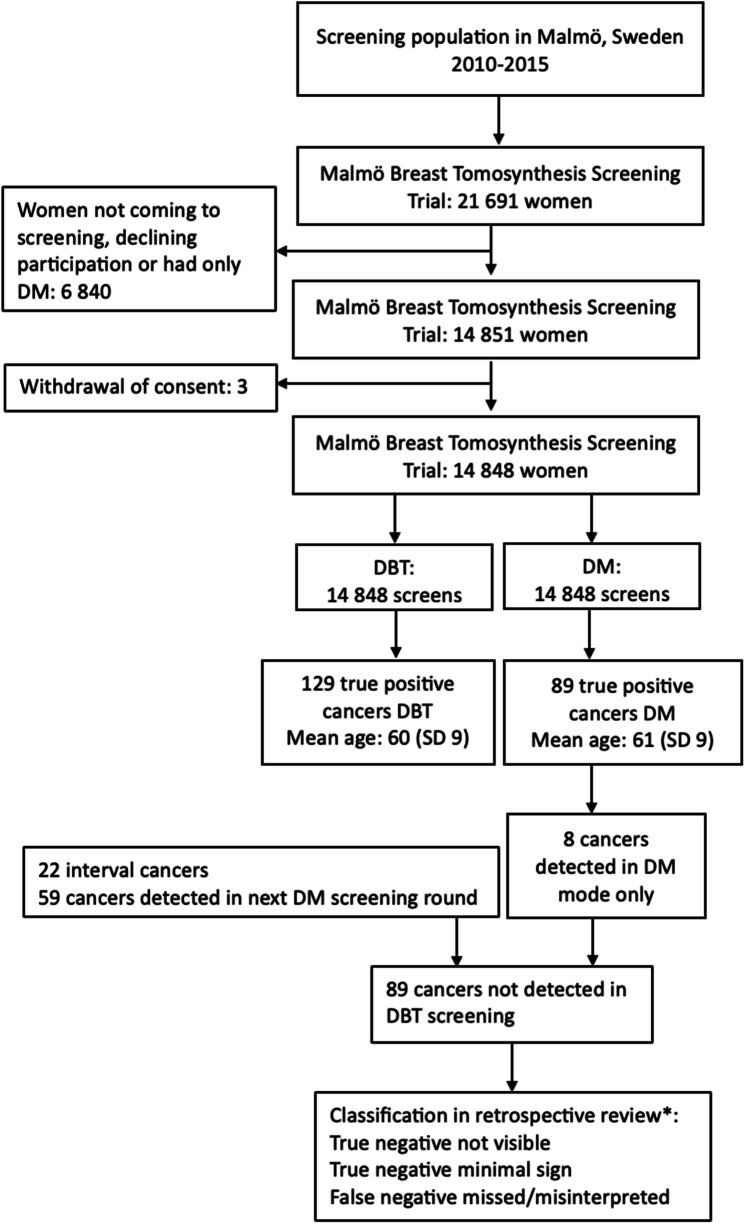
Flow chart of population and design in the Malmö Breast Tomosynthesis Screening Trial. DBT, digital breast tomosynthesis; DM, digital mammography; SD, standard deviation. *The classification was separately assessed both in relation to DBT and DM at screening in the Malmö Breast Tomosynthesis Screening Trial

In total, 21,691 women were invited to participate in the MBTST, with 14,851 undergoing trial examination. Following three withdrawals of consent, the final number of participating women was 14,848. In total, 137 women were diagnosed with screen-detected breast cancer in the MBTST, of which 129 were diagnosed in the DBT reading mode. There were, in total, 89 cancers not detected with DBT in the MBTST; eight cancers were detected solely in the DM reading mode, 59 were diagnosed during subsequent DM screening rounds, and 22 were interval cancers. Since the eight cancers detected solely with DM were DM true positives and therefore had no subsequent imaging for review, there were 81 cancers available for assessment of true/false negatives based on DM. The mean age of women at the time of cancer diagnosis, not detected during MBTST screening, was 61 years (standard deviation (SD) 9) and 60 years (SD 9) for those whose cancers were detected at DBT in the MBTST (Fig. 1).

### Retrospective review of images

The proportion of cancers classified as false negative in DBT non-detected cancers was 25% (22/89) based on the review of DBT at screening and 17% (14/81) based on the review of DM at screening. The proportion of false negatives categorized as misinterpreted in false negatives was 18% (16/89) based on DBT and 15% (12/81) based on DM. The overall false negative rate in DBT screening was 1.5 per 1000 screened women (22/14,848, 95% confidence interval 0.9−2.2). Forty-six cancers (52%) were classified as true negative non-visible and 21 (24%) as true negative minimal sign in relation to DBT compared with 45 (56%) and 22 (27%) in relation to DM, respectively (Table 1).

**Table 1:** Retrospective classification of cancers not detected in digital breast tomosynthesis (DBT) screening, in relation to DBT and digital mammography (DM) images at screening. Numbers within parentheses are percentages

Parameters	Cancers not detected, total	Interval cancers	Cancers in first DM round	Cancers detected in DM group only
**Classification based on DBT**				
True negative	67 (75)	17 (77)	44 (78)	4 (50)
Non-visible	46 (52)	11 (50)	33 (56)	2 (25)
Minimal sign	21 (24)	6 (27)	13 (22)	2 (25)
False negative	22 (25)	5 (23)	13 (23)	4 (51)
Missed	6 (7)	0	5 (9)	1 (13)
Misinterpreted	16 (18)	5 (23)	8 (14)	3 (38)
Total	89 (100)	22 (100)	59 (100)	8 (100)
**Classification based on DM**				
True negative	67 (83)	15 (68)	52 (88)	n/a
Non-visible	45 (56)	12 (55)	33 (56)	n/a
Minimal sign	22 (27)	3 (14)	19 (32)	n/a
False negative	14 (17)	7 (32)	7 (12)	n/a
Missed	2 (3)	1 (5)	1 (2)	n/a
Misinterpreted	12 (15)	6 (27)	6 (10)	n/a
Total	81 (100)	22 (100)	59 (100)	n/a

The most common reason for non-detection in false negative cancers in DBT screening was a normal-appearing density, 50% (11/22), followed by normal-appearing calcifications, which represented 18% (4/22). The most common reason in cancers classified as true negatives minimal sign was a finding deemed too small, 52% (11/21) (Table 2).

**Table 2:** Reasons for non-detection in false negative and true negative minimal sign breast cancers in digital breast tomosynthesis (DBT) screening

Parameters	True negatives—minimal sign	False negatives	Total
Too small finding	11 (52)	0	11 (26)
Dense tissue	0	2 (9)	2 (5)
Busy breast	3 (14)	2 (9)	5 (12)
Normal-appearing density	3 (14)	11 (50)	14 (33)
Normal-appearing calcifications	3 (14)	4 (18)	7 (16)
Post-surgery changes	1 (5)	2 (9)	3 (7)
Other	0	1^a^ (5)	1 (2)
Total	21 (100)	22 (100)	43 (100)

^a^ Case was judged as a mistake by radiologists, i.e., no specific reason was identified

**Fig. 2:**
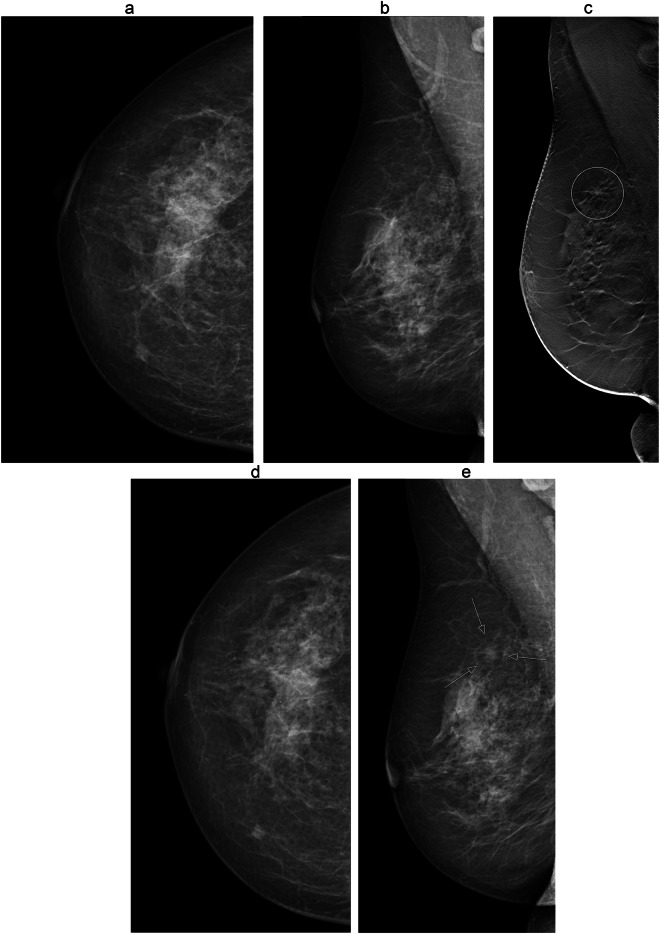
64-year-old woman who participated in the Malmö Breast Tomosynthesis Screening Trial. Images at screening; craniocaudal (**a**) and mediolateral oblique view (**b**) and tomosynthesis mediolateral oblique view (**c**). She was diagnosed with a 6 mm grade 1 tubular carcinoma at the next screening round, 24 months later. Mammography images at the next screening; craniocaudal (**d**) and mediolateral oblique view with a lesion (arrows) (**e**). The cancer was retrospectively reviewed as a false negative at tomosynthesis (circle) with a radiographic appearance of spiculated density and a true negative at mammography. The reason for non-detection was a benign-appearing density

**Fig. 3:**
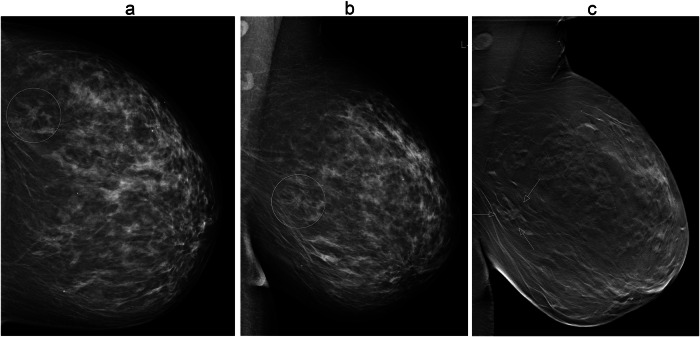
71-year-old woman who participated in the Malmö Breast Tomosynthesis Screening Trial. She was diagnosed with a 12 mm breast cancer of no special type. The cancer was detected at digital mammography only. Images at screening; digital mammography in craniocaudal and mediolateral views (**a**, **b**), findings in circles. One-view digital breast tomosynthesis at screening (**c**) with lesion (arrows), retrospectively classified as false negative by reviewers. The reason for non-detection at digital breast tomosynthesis was a busy breast

The most common dominant radiographic appearance of false negative cancers in DBT screening, as assessed at DM at diagnosis, was a spiculated density, 41% (9/22), followed by circumscribed/indistinct density, 18% (4/22) and calcifications, 18% (4/22). Figures 2 and 3 provide image examples of false-negative cancers. In true negatives minimal sign, the most common radiographic appearance was calcifications, 48% (10/21), and in true positives, the most common radiographic appearance was a spiculated density, 68% (88/129). The proportion of spiculated densities was lower in false negatives compared with true positives, *p* = 0.01.

### Tumor characteristics and breast density

False negatives occurred in 36% (8/22) in women with high density, compared with 58% (39/67) of true negatives and 61% (78/129) of true positives. The proportion of high breast density cases was lower in false negatives compared with true positives, *p* = 0.04 (Tables 3 and 4).

**Table 3:** Radiographic appearances of breast cancers at diagnosis in the Malmö Breast Tomosynthesis Screening Trial

Radiographic appearances	False negatives	True negatives	True positives	Total	*p*-value^a^
Spiculated density	9 (41)	2 (3)	88 (68)	28 (13)	0.01
Circumscribed/indistinct density	4 (18)	7 (10)	18 (14)	99 (45)	
Calcifications	4 (18)	10 (15)	17 (13)	32 (15)	
Architectural distortion	3 (14)	0	5 (4)	8 (4)	
Asymmetry	1 (5)	2 (3)	0	4 (2)	
Other	1 (5)	2 (3)	0	3 (1)	
Mammographically occult	0	44 (67)	1 (1)	44 (20)	
Total	22 (100)	67 (100)	129 (100)	218 (100)	

^a^ Comparing proportions of false negatives with true positives

**Table 4:** Breast density and tumor characteristics of breast cancers in the Malmö Breast Tomosynthesis Screening Trial

Parameters	False negatives	True negatives	True positives	Total	*p*-value^a^
Age, mean (SD)	62 (9)	62 (9)	60 (9)	61 (9)	
Cancers in total	22 (100)	67 (100)	129 (100)	218 (100)	
Breast density					
High	8 (36)	39 (58)	78 (61)	125 (57)	0.04
Low	14 (64)	28 (42)	51 (40)	93 (43)	
Type and size					
DCIS	4 (18)	21 (31)	17 (13)	42 (19)	
Invasive	18 (82)	46 (69)	112 (87)	176 (81)	0.24
Size, mean (SD)	13 (8)	15 (10)	14 (10)	14 (10)	
Histological type					
No special type	10 (46)	38 (57)	71 (55)	119 (55)	0.41
ILC	5 (23)	7 (10)	23 (18)	35 (16)	
DCIS	4 (18)	21 (31)	17 (13)	42 (19)	
Tubular	1 (5)	1 (2)	17 (13)	19 (9)	
Others	2 (9)^b^	0	1 (1)^c^	3 (1)	
Molecular subtypes					
Luminal A	4 (24)	10 (22)	54 (49)	68 (39)	0.053
Non-luminal A	13 (77)	36 (78)	57 (51)	106 (61)	
Missing	1		1	2	

*SD* standard deviation, *DCIS* ductal carcinoma in situ, *ILC* invasive lobular carcinoma

^a^ Comparing proportions of false negatives with true positives

^b^ Bone metaplastic cancer and one micropapillary mucinous cancer

^c^ Cancer detected as lymph node recurrence

The mean size of breast cancer tumors showed no difference across false negatives (13 mm, SD 8), true negatives (15 mm, SD 10), and true positives (14 mm, SD 10). There was no difference in the proportion of invasive cancers in false negatives and true positives (82% (18/22) vs. 87% (112/129), *p* = 0.53). The most common histological cancer type was no special type in all groups: 46% (13/22) in false negatives, 57% (38/67) in true negatives, and 55% (71/129) in true positives. The proportion of invasive lobular carcinomas was 23% (5/22) in false negatives compared with 10% (7/67) in true negatives and 18% (23/129) in true positives, whereas the proportion of ductal carcinoma in situ was 18% (4/22) in false negatives, 31% (21/67) in true negatives and 13% (17/129) in true positives (Table 4). Moreover, there was a difference in the proportion of luminal A-like cancers among invasive false-negative and true-positive cancers (24% (4/17) vs. 49% (54/111)), but the study was not powered for subgroup analyses (*p* = 0.053).

## Discussion

DBT is a more sensitive screening method than digital mammography (DM). However, there are still cancers that remain undetected. This retrospective informed consensus review of non-detected cancers in the Malmö Breast Tomosynthesis Screening Trial (MBTST) classified 22 out of 89 (25%) cancers not detected in one-view DBT screening as false negatives—either misinterpreted or missed—based on DBT images at screening. The proportion of false negative cancers based on DM at screening was marginally lower, accounting for 14 out of 81 cancers (17%). A predominant reason behind false negative and minimal sign cancers in DBT screening was the presence of a normal-appearing density, representing 34% (14/43) of cases. High breast density was less common among false negative cancers. Furthermore, the incidence of spiculated densities was lower compared to true-positive cancers in DBT screening. Our findings suggest a slightly heightened retrospective visibility in DBT screening compared to DM screening for non-detected cancers, underscoring crucial learning points in DBT screen interpretation.

Our study shows that there is a net increase in screen-detected cancers in DBT screening, with false negatives considered, compared with DM screening, which further adds to the benefits of DBT screening. But to date, there remains a scarcity of studies examining false negative cancers in DBT screening. A retrospective independent review of screen-detected cancers in the Oslo Breast Tomosynthesis Screening Trial, incorporating false-negative scores from one of two readers and interval cancers, showed a non-detection rate of 31% in DBT screening and 30% in DM screening, diverging from our findings [6]. The Oslo study, however, had a different study design than our study and did not include screen-detected cancers in the subsequent DM round in their review. A recent study on non-detected cancers in the randomized controlled Norwegian To-Be DBT trial 1 performed blinded and informed consensus reviews, using both interval cancers and cancers in the next round (*n* = 110) [12]. They showed a false negative rate of 16.4% in an open consensus review, which is lower than ours (25%), but if significant minimal signs from that study are considered false negatives, in line with our definition, the rate is more similar, 30.9%. A previous study on DM also used both interval cancers and screen-detected cancers as potential false negatives. Hoff et al classified 33% of IC and 20% of screen-detected as false negatives in a consensus review by four radiologists, similar to our results [13].

Given the inherent variations in the definitions in retrospective reviews of false negatives, comparing them directly becomes challenging. Moreover, the trials upon which these reviews are based exhibit disparities in design, encompassing different age groups among other variables, further complicating direct comparisons. Therefore, it becomes crucial to shift the focus towards extracting learning points from these reviews, particularly regarding how to enhance DBT screen interpretation by assimilating insights from misinterpretations and errors. Also, artificial intelligence (AI) has the potential to aid screen-reading to reduce the number of false negatives [14]. There is, however, so far limited evidence about AI in DBT screening [15]. The most common radiographic appearance in false negative cancers in the MBTST and in the Oslo trial was spiculated/stellate appearances [6]. However, this characteristic is also observed in true-positive lesions and, in the MBTST, in false-positive lesions as well [5, 16]. The ability of DBT to visualize spiculated features has implications for screen interpretation, especially in prevalence screening scenarios where there is no prior DBT for comparison. Thus, one might expect an improvement in the learning curve during incidence screening rounds with DBT. Previous studies have not presented the reasons behind false negative cancers in DBT screening. Benign-appearing density, the most common retrospectively assessed reason in our study, is, however, a known and common misleading appearance in DM screening [17]. The observation that false negative cancers were more common in less dense breasts in our study is probably due to visibility issues, i.e., cancers in dense breasts are less visible retrospectively and hence classified as true negatives. Furthermore, various image acquisition parameters and post-processing techniques across different vendors and systems could potentially influence both false negatives and the classification of radiographic appearances. Notably, the reconstruction algorithm utilized in the MBTST prioritized soft tissue over calcifications and that might have had an impact on our findings.

This study presents several limitations. Firstly, given the small number of non-detected cancers, with even fewer classified as false negatives, the study did not have enough power to fully demonstrate or exclude differences, and comparisons between groups should be interpreted with caution. Our utilization of a fully informed review design might have influenced the observed proportions of minimal signs and false negative cancers, compared with a blinded review [18, 19]. Additionally, not all four members of the review panel assessed all non-detected cancers, primarily due to logistical reasons, although the most experienced panel member was present at all reviews. Furthermore, the radiographic appearances and tumor characteristics of non-detected cancers in DM screening in the MBTST have been described previously [2, 7] but were not evaluated in terms of false negatives or reasons behind non-detection in this study focusing on DBT non-detected cancers, meaning we could not compare the false negative rates between DBT and DM. Also, the DBT examinations were performed in the mediolateral oblique view only, which could be a reason behind false negatives [20], but one should bear in mind that the 34% increase in cancer detection obtained with this imaging protocol is in line with studies including two-view DBT [3, 4, 21–23].

In conclusion, although DBT has a higher sensitivity compared with DM, cancers still remain undetected. In this study, the proportion of false negative cancers in non-detected cancers in DBT screening was slightly higher when retrospectively reviewing one-view DBT images compared with two-view DM images. False negative cancers in DBT screening often exhibited spiculated features, albeit less frequently than true-positive cancers, and were missed or misinterpreted due to benign appearances. These differences in characteristics underscore the importance of awareness of DBT-specific learning points in screen-reading practices and the potential to further optimize cancer detection in DBT screening in the future, likely also involving AI.

## Abbreviations

BI-RADS: Breast Imaging and Reporting Data System
DBT: Digital breast tomosynthesis
DM: Digital mammography
MBTST: Malmö Breast Tomosynthesis Screening Trial
SD: Standard deviation
